# Holistic Evaluation of Quality Consistency of *Ixeris sonchifolia* (Bunge) Hance Injectables by Quantitative Fingerprinting in Combination with Antioxidant Activity and Chemometric Methods

**DOI:** 10.1371/journal.pone.0148878

**Published:** 2016-02-12

**Authors:** Lanping Yang, Guoxiang Sun, Yong Guo, Zhifei Hou, Shuai Chen

**Affiliations:** 1 School of Pharmacy, Shenyang Pharmaceutical University, Shenyang, 110016, China; 2 School of Pharmacy, Fairleigh Dickinson University, Florham Park, New Jersey, 07932, United States of America; Macau University of Science and Technology, MACAO

## Abstract

A widely used herbal medicine, *Ixeris sonchifolia* (Bge.) Hance Injectable (ISHI) was investigated for quality consistency. Characteristic fingerprints of 23 batches of the ISHI samples were generated at five wavelengths and evaluated by the systematic quantitative fingerprint method (SQFM) as well as simultaneous analysis of the content of seven marker compounds. Chemometric methods, i.e., support vector machine (SVM) and principal component analysis (PCA) were performed to assist in fingerprint evaluation of the ISHI samples. Qualitative classification of the ISHI samples by SVM was consistent with PCA, and in agreement with the quantitative evaluation by SQFM. In addition, the antioxidant activities of the ISHI samples were determined by both the off-line and on-line DPPH (2, 2-diphenyl-1-picryldrazyl) radical scavenging assays. A fingerprint–efficacy relationship linking the chemical components and *in vitro* antioxidant activity was established and validated using the partial least squares (PLS) and orthogonal projection to latent structures (OPLS) models; and the online DPPH assay further revealed those components that had position contribution to the total antioxidant activity. Therefore, the combined use of the chemometric methods, quantitative fingerprint evaluation by SQFM, and multiple marker compound analysis in conjunction with the assay of antioxidant activity provides a powerful and holistic approach to evaluate quality consistency of herbal medicines and their preparations.

## Introduction

Traditional Chinese Medicine (TCM) and herbal preparations have been widely used by billions of people around the world for thousands of years. The World Health Organization (WHO) recommends chromatography finger printing as a means of identification and quality evaluation since 1991 [1]. The Chinese State Food and Drug Administration (SFDA), US Food and Drug Administration (FDA) and European Medicine Agency (EMA) have all accepted the chromatography fingerprinting method and promote its use for the quality control of herbal preparations [2–5]. SFDA began to require that all injectable preparations made from TCM or their raw materials be standardized by chromatography fingerprinting in 2000 [6]. The fingerprinting technique, especially chromatography fingerprinting, has become a powerful tool for the quality control of complex multi-component herbal preparations. Current chromatography fingerprinting methods are mainly performed on TLC, HPLC, UHPLC, GC, CE platforms [7,8]; and HPLC is preferred due to its high sensitivity, reproducibility, adaptability for a wide range of samples, and especially availability of various detectors, such as chemiluminescence detector [9] and mass spectrometric detectors [10–12]. The conventional chromatography fingerprinting methods are mostly qualitative based on simple comparison of similarity of the fingerprints, and often lack the quantitative assessment of the fingerprints. SQFM was recently developed to address the issue of quantitative comparison of the fingerprints of the reference standards and test samples [13]. In addition, unsupervised pattern recognition methods, such as PCA as well as supervised methods, such as SVM, PLS, partial least squares discriminant analysis (PLS-DA), OPLS, and orthogonal projection to latent structures discriminate analyses (OPLS-DA), have also been increasingly applied to chromatography fingerprinting analysis in support of the quality control of TCM and herbal preparations [14–17]. For example, Custers et. al. reported that PCA of fingerprints was able to distinguish the genuine medicines from the counterfeits [8]. Jian Liang et. al. adopted PCA and OPLS methods to evaluate the quality consistency of complex TCM preparations [11].

The injectable preparation of *Ixeris sonchifolia* (Bunge) Hance (*I*. *sonchifolia*), also known as “Kudiezi” in Chinese, has been widely used for its anti-inflammatory and haemostatic effects, its influence on improving blood circulation, and potential protection against ischemia brain injury with relatively low side effects for over 30 years [18,19]. The annual sale revenue has reached more than one billion Chinese YUAN in China. The chemical compositions of *I*. *sonchifolia* are quite complex and include nucleosides, phenolic acids, flavonoids, sesquiterpene lactones, triterpenes and steroids, lignans and amino acids [20–22]. Most published reports focused on only one or a limited number of components of *I*. *sonchifolia* [23,24]. In this study, the content of seven marker compounds, including two nucleosides (i.e. uridine (UR) and adenosine (AD)), three phenolic acids (i.e. chlorogenic acid (CGA), caffeic acid (CFA) and chicoric acid (CCA)), and two flavonoids (i.e. luteolin-7-β-D-glucuronide (LGR) and luteolin-7-glucoside (LG)) were determined simultaneously using a validated HPLC-DAD method. The fingerprints of 23 ISHI samples were generated at 5 different detection wavelengths using the HPLC-DAD method and systematically evaluated using both qualitative and quantitative similarity comparison. SQFM [13,25,26] can not only qualitatively evaluate the chemical composition, but also provides quantitative similarity measures for the overall contents of the herbal preparations. In addition, PCA and SVM were also employed to assist in evaluating the fingerprints of all the ISHI samples.

Published studies have shown that the neuro-protective effects of *I*. *sonchifolia* against ischemia-induced cellular injury are provided by the antioxidant components [19,27]. The activity of the antioxidants can be determined based on the scavenging effect on DPPH radicals [28]. An easy and accurate method, the DPPH radical scavenging assay has been recommended to measure the antioxidant activity of fruit and vegetable juices or extracts [29,30]. In recent years, the DPPH radical scavenging assay has also been employed to investigate the antioxidant activity of TCM and their preparations [9,10]. In this study, both off-line and on-line DPPH assays were performed to determine the antioxidant activity of the ISHI samples. Predictive models for the antioxidant activity were also established using the PLS and OPLS methods.

## The Theory of SQFM

The sample fingerprint and reference fingerprint vectors are defined as $\vec{x}=\left(x_{1},x_{2},\ldots x_{n}\right)$ and $\vec{y}=\left(y_{1},y_{2},\ldots y_{n}\right)$, where *x*_*i*_ and *y*_*i*_ are the peak areas of the component peaks in the sample fingerprint and reference fingerprint vectors, respectively. Calculating the cosine of the angle between the sample fingerprint and reference fingerprint vectors provides qualitative similarity (*S*_*F*_) as defined in Eq 1. Although the qualitative similarity factor (*S*_*F*_) can clearly reflects the degree of similarity in the chemical compositions of the sample fingerprint and reference fingerprint in terms of distribution ratio, it is biased towards the large peaks, which raises a serious question on its validity. In order to limit the influence of the large peaks and ensure an equal weight for each peak, the sample fingerprint ($\vec{x}$) and reference fingerprint ($\vec{y}$) vectors are transformed to ${\vec{P}}_{s}=\left(\frac{x_{1}}{y_{1}},\frac{x_{2}}{y_{2}},\cdots \frac{x_{n}}{y_{n}}\right)$ and ${\vec{P}}_{0}=\left(1,1,1\cdots 1\right)$, respectively. The cosine of the angle between the vectors ${\vec{P}}_{o}$ and ${\vec{P}}_{s}$ is defined as the qualitative ratio similarity ($S_{F}^{'}$), as calculated by Eq 2. Macro qualitative similarity (*S*_*m*_) can be obtained by averaging *S*_*F*_ and $S_{F}^{'}$ as shown in Eq 3. For quantitative assessment of the fingerprints, the projection of $\vec{x}$ to $\vec{y}$ is defined as projection content similarity (*C*) as calculated in Eq 4. The projection content similarity factor (*C*) can reflect the degree of similarity in the chemical compositions of the sample fingerprint and reference fingerprint in terms of the total contents, but still suffers from the bias of the large peaks over the small peaks. The quantitative similarity (*P*) is the ratio of the total content corrected by the qualitative similarity factor *S*_*F*_, as shown in Eq 5. Combining the above two quantitative properties yields macro quantitative similarity (*P*_*m*_) as defined in Eq 6, which is a measure to monitor the overall content of chemical components in the sample fingerprint. Finally, a fingerprint leveling coefficient (*α*), as defined in Eq 7, is another quantitative parameter that is able to detect the difference between sample fingerprint and reference fingerprint.

$$S_{F}=\text{cos}\;\theta =\frac{\sum_{i=1}^{n}x_{i}y_{i}}{\sqrt{\sum_{i=1}^{n}x_{i}^{2}}\sqrt{\sum_{i=1}^{n}y_{i}^{2}}}$$ (1)

$$S_{F}^{'}=\text{cos}\;\theta^{'}=\frac{\sum_{i=1}^{n}\frac{x_{i}}{y_{i}}}{\sqrt{n\sum_{i=1}^{n}{\left(\frac{x_{i}}{y_{i}}\right)}^{2}}}$$ (2)

$$S_{m}=\frac{1}{2}\left(S_{F}+S_{F}^{'}\right)=\frac{1}{2}\left(\frac{\sum_{i=1}^{n}x_{i}y_{i}}{\sqrt{\sum_{i=1}^{n}x_{i}^{2}}\sqrt{\sum_{i=1}^{n}y_{i}^{2}}}+\frac{\sum_{i=1}^{n}\frac{x_{i}}{y_{i}}}{\sqrt{n\sum_{i=1}^{n}{\left(\frac{x_{i}}{y_{i}}\right)}^{2}}}\right)$$ (3)

$$C=\frac{\sum_{i=1}^{n}x_{i}y_{i}}{\sum_{i=1}^{n}y_{i}^{2}}\times 100\%$$ (4)

$$P=\frac{\sum_{i=1}^{n}x_{i}}{\sum_{i=1}^{n}y_{i}}S_{F}\times 100\%$$ (5)

$$P_{m}=\frac{1}{2}\left(C+P\right)=\frac{1}{2}\left(\frac{\sum_{i=1}^{n}x_{i}y_{i}}{\sum_{i=1}^{n}y_{i}^{2}}+\frac{\sum_{i=1}^{n}x_{i}}{\sum_{i=1}^{n}y_{i}}S_{F}\right)\times 100\%$$ (6)

$$\alpha =\left|1-\frac{P}{C}\right|$$ (7)

SQFM combines the macro qualitative and quantitative similarity factors (*S*_*m*_ and *P*_*m*_) [13,25,26]. The quality of the TCM and herbal preparations can be assessed and classified into different grades based on the values of *S*_*m*_ and *P*_*m*_ as well as *α*, in which the evaluation criteria by SQFM are listed in Table A in S3 File, where grade 1 belongs to the best quality and grade 8 to the worst one. Based on the criteria, all *Sm*, *Pm* and *α* are used together in the rules for classification, and the final quality grade is on the basis of the worst grade. For example, if *Sm* 0.96 (grade 1), *Pm*(%) 95.6 (grade 1) and *α* 0.02 (grade 1), then quality is grade 1; if *Sm* 0.89 (grade 3), *Pm*(%) 89.5 (grade 3) and *α* 0.07 (grade 2), then quality is grade 3; if *Sm* 0.87 (grade 3), *Pm*(%) 104.6 (grade 1) and *α* 0.25 (grade 5), then quality is grade 5.

## Materials and Methods

### Materials and reagents

**Fig 1:**
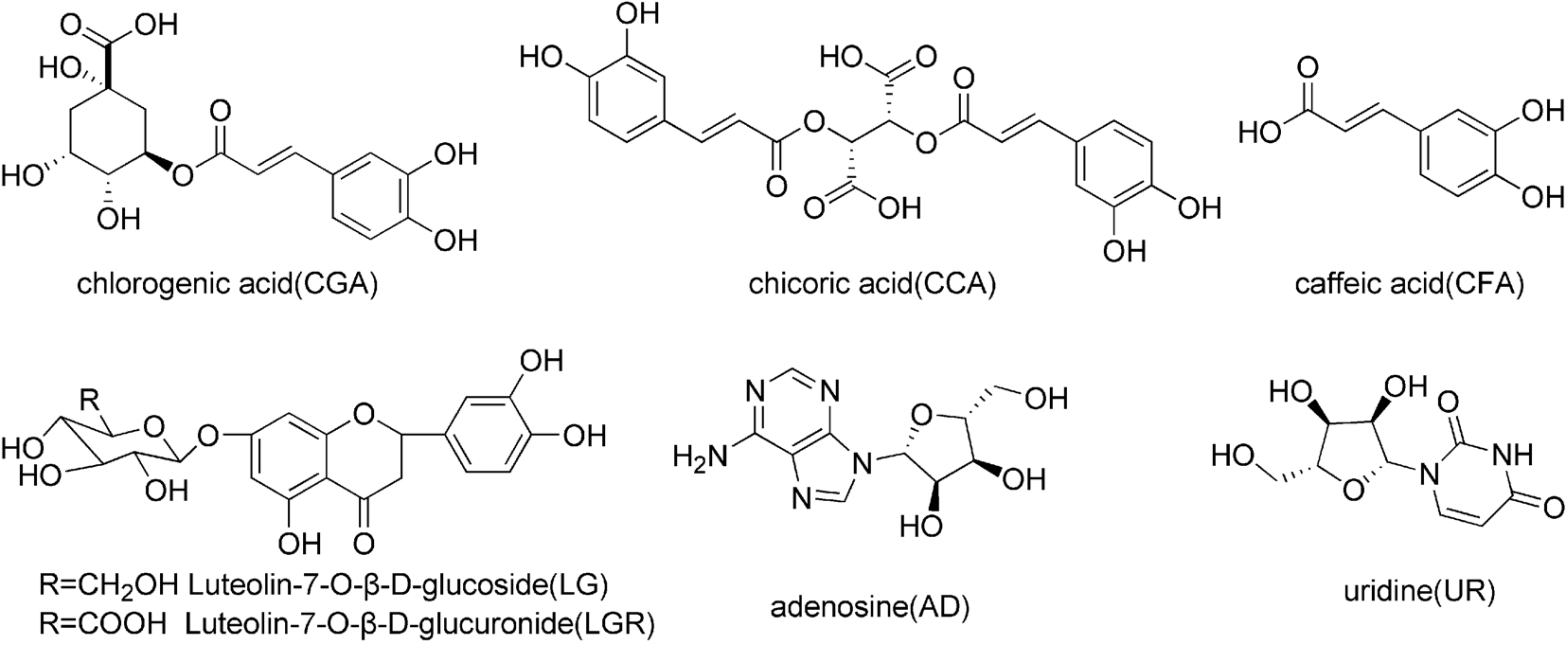
The chemical structures of seven marker compounds.

A total of 23 batches of ISHI injectable preparations (20ml, apparent concentration = 1.0g/ml), all manufactured by Shenyang Shuangding Pharmaceutical Co., Ltd., were obtained from different pharmacies in Shenyang, China. UR standard was purchased from Sigma Chemical Co. (St. Louis, MO, US). The standards of AD, CGA and CFA were acquired from the National Institute for the Control of Pharmaceutical and Biological Products (Beijing, China). The standards of CCA and LGR were supplied by Chengdu Puri France Science and Technology Development Co., Ltd. (Chengdu, China). LG standard was provided by Shanghai Winherb Medical Technology Co., Ltd. (Shanghai, China). All the standard compounds have purity above 98%. The structures of the marker compounds are shown in Fig 1.

Methanol (HPLC grade) and acetonitrile (HPLC grade) were purchased from Yuwang Industry Co., Ltd. (Shandong, China), and glacial acetic acid (HPLC grade) from Kermel Chemistry Reagent Co., Ltd. (Tianjin, China). De-ionized water and other reagents were of analytical grade.

### Equipment and chromatographic conditions

HPLC analysis was performed on an Agilent 1100 HPLC system comprised of an online degasser, a low pressure mix quaternary pump, an auto-sampler, and a diode array detector (DAD), and controlled by a ChemStation workstation (Agilent Technology, California, USA). The chromatographic separation was carried out on an Arcus EP-C18 column (250 mm × 4.6 mm, 5 μm) from Exformma Technologies (Shanghai, China). The off-line antioxidant activity assay was performed on a 722S spectrophotometer (Shanghai Precision Instrument Co., Ltd., Shanghai, China).

The mobile phase was composed of an aqueous solution containing 5 mM citric acid and 10 mM sodium dihydrogen phosphate (A) and acetonitrile containing 1.0% (v/v) glacial acetic acid (B). The gradient elution program was as follows: 0–3% B at 0–5 min, 3–7% B at 5–10 min, 7–10% B at 10–16 min, 10–17% B at 16–25 min, 17–19% B at 25–30 min, 19–20% B at 30–40 min, 20–25% B at 40–60 min. The flow rate was set at 1.0 ml/min and the injection volume was 10 μL. The column temperature was maintained at 35°C. Online UV spectra were obtained over a wavelength range of 190–600 nm.

### Preparation of standard and sample solutions

The reference standards of the marker compounds (UR, AD, CGA, CFA, CCA, LGR and LG) were accurately weighed separately and dissolved in methanol, then diluted with methanol to appropriate concentration ranges for the calibration curves, and stored at 4°C prior to use.

The ISHI samples (20ml, apparent concentration = 1.0g/ml) were filtered through 0.45 μm Millipore filters (Beijing Sunrise T&D Company, China) prior to use.

### Antioxidant activity assay

#### Off-line DPPH assay

The DPPH radical stock solution was prepared in methanol (1 mM) immediately before the experiments and protected from light. DPPH free radical scavenging capacity was determined by a decrease in the absorption at 517 nm upon reduction by an antioxidant. The DPPH assay was performed according to Pamita Bhandari et al. [31] with slight modification. Briefly, a 0.127 mM DPPH solution was prepared in methanol and 2 mL of this solution was added to 2 mL of the ISHI sample solution diluted in methanol to various concentrations (apparent concentration = 1–6 mg/mL). These solutions were allowed to stand in dark for 40 minutes and the absorbance was measured at 517 nm against a blank. All tests were performed in triplicates. The radical scavenging capacity is expressed as percent inhibition and calculated using the following equation: %inhibition = [(*A*_*control*_ − *A*_*sample*_) / *A*_*control*_]×100, where *A*_*control*_ is the absorbance of the negative control and *A*_*sample*_ is the absorbance at the presence of the ISHI sample. The percent inhibition was plotted against the sample concentration in order to calculate *IC*_50_ values (the concentration of samples required to scavenge 50% of DPPH radicals).

#### On-line HPLC-DAD-DPPH assay

This on-line assay was performed by using the method introduced by Jyh-Horng Wu et al. [32] with slight modifications. The ISHI solution (11 μL) was injected into the HPLC system (see section ‘Equipment and chromatographic conditions‘) at a flow rate of 0.8 mL/min. The chemical components in the ISHI solution was separated and detected at 260 nm. The eluted compounds reached to a reaction coil (5000 mm × 0.007/0.18 mm i.d. PEEK tubing from Agilent), where the 0.127 mM methanol DPPH solution was delivered via another LC pump (Iso pump, Agilent 1100 series) at a flow rate of 0.3 mL/min. After the eluent mixed with DPPH solution, negative peaks were detected at 517 nm.

### Chemometric analysis

#### SVM

SVM is an efficient method for classification and is widely used on disease diagnosis or medical assistance [33, 34]. In this study, SVM was adopted to classify the ISHI samples based on RBF kernel type using SPSS statistic software (SPSS Clementine 12.0, SPSS Inc., USA). The training dataset was 23 samples of (x_i_, y_i_), where *x*_*i*_ is a feature vector of seven markers’ contents in a d-dimensional feature space R^d^ and y_i_ ∈ {−1, +1}, y = −1 represents the integrated grade ≤2; y = +1 represents grade>2.

#### PCA

PCA is used to qualitatively analyze the samples by reducing the number of variables and data dimensionality. The score plot of PCA is a map of the observations that shows the possible presence of any outliers in the data [14,15]. In this study, PCA analysis was performed on 49 common peaks detected in all the ISHI samples using SIMCA-P+ software (Version 13.0, Umetrics, Umea, Sweden), and the significance level was set at 95%.

#### PLS and OPLS analysis

PLS method is a versatile linear regression algorithm that can be used to predict either the continuous or discrete/categorical variables; and OPLS (another linear regression method) is an extension of PLS, which reduces the complexity of models while preserves the ability of prediction by removing descriptor variables *X* (data set) that is not correlated (i.e. orthogonal) to property variables *Y* (response set) [16, 35]. In this study, both PLS and OPLS models were constructed to characterize the correlation between the total antioxidant activity and the chemical content of the ISHI samples using the areas of 49 characteristic peaks as the descriptor matrix *X* and the 1/IC_50_ values as the response matrix *Y* with the SIMCA-P+ software (Version 13.0, Umetrics, Umea, Sweden). The confidence level was set at 95%.

## Results and Discussion

### Optimization of chromatographic conditions and method validation

In order to achieve reproducible separation and acceptable resolution in a short analysis time, we investigated four mobile phase (MP: MP1**~**MP4) conditions and three gradient elution programs (GEP: GEP1**~**GEP3). The index of the fingerprints information amount (*I*) [36], which represents the signal size, signal homogenization and the information amount, was adopted to optimize the mobile phase condition and gradient program. From S1 File, it was found that the *I* values for the four mobile phase conditions MP1**~**MP4 are 15.7, 14.9, 15.9 and 15.5, respectively; while the *I* values for the three gradient programs GEP1**~**GEP3 are 13.6, 16.4 and 16.7, respectively. Therefore, the mobile phase condition MP3 (*I* = 15.9) and gradient program GEP3 (*I* = 16.7) were selected as the optimized conditions.

The calibration curves were established by plotting the peak area against the concentration of each standard marker compound in the concentration range suitable for the expected concentration of the marker compounds in the ISHI samples. Table 1 summarizes the linearity results and the method shows acceptable linearity (R^2^≥0.9996) for all the marker compounds in the targeted concentration ranges. The limit of detection (LOD, S /N = 3) and the limit of quantification (LOQ, S /N = 10) were also determined to be in the range of 0.10–0.37 μg·mL^-1^ and 0.41–1.63 μg·mL^-1^ for the marker compounds. The system repeatability was evaluated by analyzing six individual mixed standard solutions; the stability of the sample solution was validated by analyzing a single standard mixture solution stored at room temperature for 0, 2, 4, 8, 16, and 24 h, respectively; intra-day and inter-day precision of the method were evaluated by nine replicate injections of the standard mixture solution three times a day over three consecutive days. The relative standard deviation (RSD%) values for the repeatability, stability, intra-day and inter-day precision were all less than 0.4% and 2.2% for the relative retention time and the peak area of the seven marker standards, respectively. The recovery was validated by a standard spiking test, and the average recovery values for the marker standards were between 96.4% and 108.3% with RSD% less than 3.1%, suggesting that the method was accurate. Method validation demonstrated that the method was precise, accurate and sensitive enough for simultaneously quantitative analysis of the seven marker compounds in ISHIs.

**Table 1:** Results for linearity, LOD and LOQ of seven marker compounds.

Compound^a^	Calibration curve^b^	R^2^	LOD(μg·mL^-1^)	LOQ(μg·mL^-1^)	Linear range(μg·mL^-1^)
UR(265nm)	*y* = 33.41*x*+16.38	0.9998	0.15	0.58	0.63–6.30
AD(260nm)	*y* = 11.41*x*+15.28	0.9998	0.37	1.63	1.92–19.2
CGA(330nm)	*y* = 21.28*x*+59.83	0.9997	0.15	0.58	1.20–24.0
CFA(330nm)	*y* = 30.76*x*+78.35	0.9997	0.10	0.41	1.80–36.0
CCA(335nm)	*y* = 29.94*x*+123.6	0.9997	0.11	0.60	6.60–132
LGR(350nm)	*y* = 18.78*x*+30.65	0.9998	0.26	0.98	14.3–360
LG(350nm)	*y* = 20.95*x*+88.50	0.9996	0.20	0.90	3.00–30.0

^a^UR, Uridine; AD, Adenosine; CGA, Chlorogenic acid; CFA, Caffeic acid; CCA, Cichoric acid; LGR, Luteolin-7-β-D-glucuronide; LG, Luteolin-7-glucoside.

^b^*y* is the peak area, *x* is the concentration injected (μg·mL^-1^).

### Fingerprinting and marker compound analysis by HPLC-DAD

**Fig 2:**
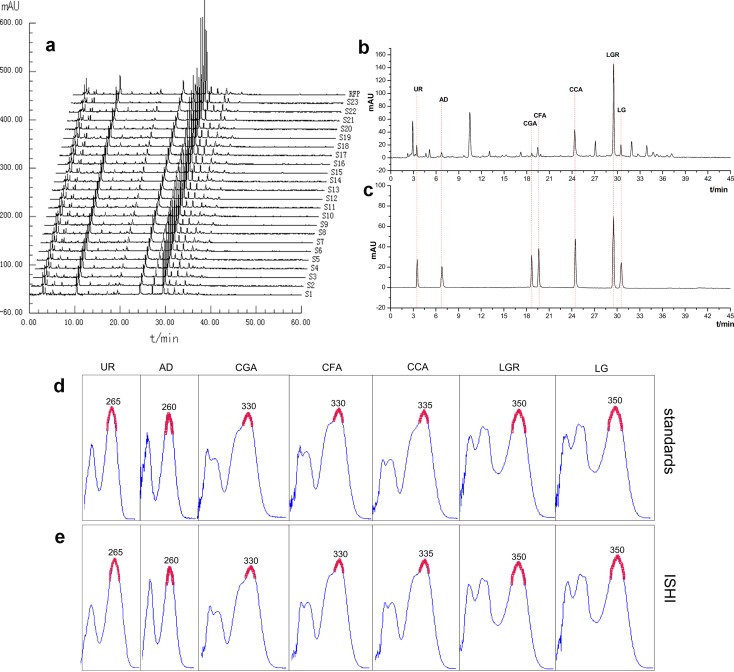
The chromatograms and UV absorption spectra of ISHI and standards: (a) The chromatograms of 23 batches of ISHI samples at 260 nm. (b), (c) The chromatograms of ISHI and mixed standards, respectively. (d), (e) The UV absorption spectra of standards and ISHI, respectively.

The marker compounds show very different UV absorption as shown in Fig 2D. It is reasonable to believe that the components in the ISHI samples also have different absorption behavior. Therefore, the fingerprints of the ISHI samples were generated at five different wavelengths (i.e., 260 nm, 265 nm, 330 nm, 335 nm and 350 nm) corresponding to the absorption maxima of the marker compounds in order to capture as many peaks as possible. A total of 49, 46, 38, 39 and 33 peaks common to all the ISHI samples were identified at 260 nm, 265 nm, 330 nm, 335 nm and 350 nm, respectively. Fig 2A shows the overlay chromatograms of 23 ISHI samples at 260 nm and the representative chromatograms of the sample and the marker compounds are shown in Fig 2B and 2C, respectively. The reference fingerprint (RFP) was generated by averaging all the sample chromatograms.

The content of the marker compounds was simultaneously determined in all 23 batches of the ISHI samples using the established calibration curves (Table 1). The quantitative information of the marker compounds are presented in Table 2. CCA and LGR were found to be the main components in all the samples with the average values of 68.9 and 97.1 mg/L, respectively. A large variation in the content of AD and LG was observed in all the samples as reflected by relative standard deviation (%RSD) of 53.1% for AD and 47.7% for LG, respectively. The high %RSD was due to the undetectable and very low levels of AD and very high levels of LG in Sample 21 (S21) and 23 (S23). It was noted that S21 and S23 were significantly different from the other samples in the content of other marker compounds: the content of AD, CGA, CFA and CCA were obviously lower, and the content of LGR and LG higher in S21 and S23 than the other samples.

**Table 2:** Overview of the contents of seven marker compounds and the IC_50_ values for 23 batches of ISHIs.

			Content (mg·L^-1^)					
Sample	UR	AD	CGA	CFA	CCA	LGR	LG	IC50 (mg·mL^-1^)
S1	3.31	3.40	10.32	14.78	58.30	74.63	5.63	4.01
S2	2.73	8.31	7.85	9.57	60.23	75.30	5.72	4.24
S3	2.73	6.48	6.51	15.07	62.37	83.23	4.56	4.52
S4	2.45	5.27	7.07	15.84	64.69	88.41	5.34	4.94
S5	2.55	6.16	6.13	14.84	62.31	82.88	4.59	4.11
S6	2.17	5.65	6.49	15.94	60.82	81.69	4.75	4.12
S7	2.18	2.30	7.93	19.44	76.98	86.10	6.50	4.40
S8	3.53	3.92	8.73	22.88	90.60	89.30	6.51	4.61
S9	2.98	1.76	7.81	17.09	71.73	71.42	4.72	4.64
S10	3.91	9.49	9.22	16.92	61.29	81.36	4.34	4.34
S11	3.19	2.20	8.34	18.62	81.98	86.66	6.74	4.28
S12	3.05	2.48	8.03	18.00	74.36	79.01	5.93	4.70
S13	3.16	3.71	8.33	18.21	74.46	94.52	8.17	4.27
S14	2.91	3.95	8.79	15.36	66.27	108.74	15.90	4.26
S15	3.63	4.67	8.04	21.51	77.63	110.90	8.83	4.40
S16	3.86	5.63	8.09	23.21	78.81	109.19	9.22	3.96
S17	3.91	3.46	7.98	22.55	82.42	116.00	8.95	4.45
S18	3.32	4.59	8.18	19.91	89.29	95.93	7.17	3.90
S19	3.80	4.94	7.57	21.94	75.69	104.23	8.21	3.81
S20	2.38	5.56	6.51	15.44	62.77	82.63	4.48	4.57
S21	2.22	0.06	1.55	7.44	40.97	145.19	14.64	5.48
S22	3.58	5.28	10.07	18.41	65.67	135.37	14.99	3.48
S23	2.28	ND^a^	2.80	3.28	46.06	150.95	15.22	5.55
Mean	3.04	4.31	7.49	16.79	68.94	97.11	7.87	4.35
RSD(%)	19.60	53.10	26.50	29.10	17.90	23.00	47.70	13.30
	Percent content (%, *m*/*m*)							
Sample	UR	AD	CGA	CFA	CCA	LGR	LG	***P***_7C_
S1	109.0	78.9	137.8	88.0	84.6	76.9	71.6	92.4
S2	89.9	192.7	104.8	57.0	87.4	77.5	72.7	97.4
S3	89.9	150.2	86.9	89.7	90.5	85.7	57.9	93.0
S4	80.6	122.2	94.4	94.3	93.8	91.0	67.8	92.0
S5	83.9	142.7	81.8	88.4	90.4	85.4	58.3	90.1
S6	71.4	130.9	86.6	94.9	88.2	84.1	60.3	88.1
S7	71.8	53.3	105.9	115.8	111.7	88.7	82.5	89.9
S8	116.3	90.9	116.5	136.3	131.4	92.0	82.7	109.4
S9	98.2	40.9	104.2	101.8	104.0	73.5	60.0	83.2
S10	128.7	219.9	123.1	100.8	88.9	83.8	55.1	114.3
S11	105.2	51.1	111.3	110.9	118.9	89.2	85.6	96.0
S12	100.6	57.6	107.1	107.2	107.9	81.4	75.3	91.0
S13	104.1	86.0	111.2	108.5	108.0	97.3	103.8	102.7
S14	95.9	91.5	117.3	91.5	96.1	112.0	202.0	115.2
S15	119.4	108.2	107.4	128.1	112.6	114.2	112.1	114.6
S16	127.2	130.5	108.0	138.2	114.3	112.4	117.1	121.1
S17	128.7	80.3	106.5	134.3	119.5	119.5	113.6	114.6
S18	109.3	106.5	109.2	118.5	129.5	98.8	91.0	109.0
S19	125.1	114.5	101.0	130.7	109.8	107.3	104.2	113.2
S20	78.5	128.9	86.8	92.0	91.1	85.1	56.9	88.5
S21	73.2	1.5	20.7	44.3	59.4	149.5	186.0	76.4
S22	118.1	122.4	134.3	109.6	95.3	139.4	190.4	129.9
S23	75.0	ND^a^	37.3	19.5	66.8	155.4	193.3	78.2
Mean	100.0	100.0	100.0	100.0	100.0	100.0	100.0	100.0
RSD(%)	19.6	53.1	26.5	29.1	17.9	23.0	47.7	14.5

^a^Not detected.

### Fingerprint evaluation by SQFM

The fingerprints of the ISHI samples generated at 5 different wavelengths (260 nm, 265 nm, 330 nm, 335 nm and 350 nm) were evaluated using the SQFM, in which we averaged the 23 batches of sample fingerprints to give the reference fingerprints under each wavelength, respectively. The macro qualitative and quantitative similarity factors (*S*_*m*_ and *P*_*m*_) as well as the leveling coefficient (*α*) were computed by importing the fingerprint signals of the sample fingerprint and reference fingerprint into an in-house developed software “Digitized Evaluation System for Super-Information Characteristics of TCM-CFPs 4.0” (software certificate No. 0407573, China). A separate set of integrated *S*_*m*_, *P*_*m*_ and *α* values ($S_{m}^{'}$, $P_{m}^{'}$ and *α*') was also calculated according to Eqs 8–10 to avoid potential bias of different wavelengths. The calculated similarity factors and leveling coefficients for all the samples are presented in Table 3.

$$S_{m}^{'}=\sqrt{\frac{1}{5}\sum_{i=1}^{n}S_{m_{i}}^{2}}$$ (8)

$$P_{m}^{'}=\sqrt{\frac{1}{5}\sum_{i=1}^{n}P_{m_{i}}^{2}}$$ (9)

$$\alpha^{'}=\sqrt{\frac{1}{5}\sum_{i=1}^{n}\alpha_{i}^{2}}$$ (10)

**Table 3:** Evaluation results for 23 batches of ISHIs by SQFM.

*Λ*	Para.	S1	S2	S3	S4	S5	S6	S7	S8	S9	S10	S11	S12
260nm	*S*_*m*_	0.89	0.98	0.96	0.96	0.97	0.97	0.98	0.89	0.97	0.90	0.98	0.97
	*P*_*m*_(%)	89.5	88.3	91.1	95.6	92.2	91.0	94.0	102.6	82.9	94.9	94.8	89.0
	*α*	0.07	0.07	0.01	0.02	0.01	0.03	0.03	0.06	0.07	0.10	0.05	0.06
	Grade	3	3	2	1	2	2	2	3	3	3	2	3
265nm	*S*_*m*_	0.91	0.96	0.97	0.96	0.97	0.97	0.97	0.97	0.96	0.97	0.97	0.97
	*P*_*m*_(%)	91.1	87.0	87.0	92.4	96.7	91.1	89.6	102.9	83.3	95.1	94.4	88.3
	*α*	0.10	0.07	0.07	0.02	0.01	0.03	0.02	0.05	0.05	0.12	0.03	0.04
	Grade	3	3	3	2	1	2	3	2	3	3	2	3
330nm	*S*_*m*_	0.97	0.97	0.97	0.98	0.98	0.99	0.98	0.98	0.97	0.99	0.98	0.99
	*P*_*m*_(%)	94.6	94.4	98.3	103.0	98.9	97.6	99.1	110.9	91.3	98.4	100.0	95.3
	*α*	0.01	0.03	0.05	0.05	0.05	0.05	0.01	0.02	0.01	0.01	0.01	0.00
	Grade	2	2	2	1	1	1	1	3	2	1	1	1
335nm	*S*_*m*_	0.97	0.96	0.97	0.98	0.97	0.98	0.98	0.98	0.96	0.97	0.98	0.98
	*P*_*m*_(%)	94.4	93.9	98.0	102.6	97.6	96.4	97.7	111.5	90.5	96.9	100.2	94.3
	*α*	0.01	0.02	0.05	0.05	0.05	0.05	0.00	0.02	0.01	0.01	0.00	0.01
	Grade	2	2	2	1	1	1	1	3	2	1	1	2
350nm	*S*_*m*_	0.97	0.97	0.98	0.94	0.97	0.97	0.98	0.97	0.98	0.98	0.97	0.98
	*P*_*m*_(%)	89.5	90.1	93.2	99.2	93.9	93.2	97.0	105.0	86.1	93.3	96.3	92.2
	*α*	0.03	0.02	0.02	0.01	0.01	0.02	0.01	0.01	0.01	0.02	0.01	0.02
	Grade	3	2	2	2	2	2	1	2	3	2	1	2
Integrated	*S*_*m*_′	0.94	0.97	0.97	0.96	0.97	0.97	0.98	0.96	0.97	0.96	0.98	0.97
	*P*_*m*_′	91.8	90.8	93.6	98.6	95.9	93.9	95.5	106.7	86.9	95.7	97.2	91.9
	*α*′	0.06	0.05	0.05	0.03	0.03	0.03	0.02	0.04	0.04	0.07	0.03	0.03
	Grade	2	2	2	1	1	2	1	2	3	2	1	2
*λ*	Para.	S13	S14	S15	S16	S17	S18	S19	S20	S21	S22	S23	RFP
260nm	*S*_*m*_	0.98	0.97	0.98	0.98	0.97	0.98	0.98	0.97	0.87	0.96	0.89	1.00
	*P*_*m*_(%)	98.5	105.1	111.1	109.5	114.1	103.2	107.2	92.4	104.6	120.0	110.2	100.0
	*α*	0.01	0.06	0.01	0.00	0.03	0.03	0.00	0.01	0.25	0.09	0.24	0.00
	Grade	1	2	3	2	3	1	2	2	5	3	5	1
265nm	*S*_*m*_	0.98	0.97	0.97	0.97	0.98	0.97	0.97	0.96	0.86	0.96	0.86	1.00
	*P*_*m*_(%)	97.8	104.9	111.6	109.1	114.8	103.5	106.4	91.6	107.9	116.8	113.5	100.0
	*α*	0.01	0.05	0.02	0.02	0.03	0.03	0.00	0.01	0.24	0.09	0.24	0.00
	Grade	1	1	3	2	3	1	2	2	5	3	5	1
330nm	*S*_*m*_	0.98	0.98	0.99	0.99	0.99	0.99	0.99	0.98	0.87	0.97	0.88	1.00
	*P*_*m*_(%)	99.1	107.0	105.4	105.8	109.6	109.5	101.9	98.7	72.6	110.7	74.7	100.0
	*α*	0.02	0.02	0.03	0.03	0.02	0.01	0.03	0.05	0.05	0.00	0.06	0.00
	Grade	1	2	2	2	2	2	1	1	5	3	5	1
335nm	*S*_*m*_	0.99	0.96	0.99	0.99	0.98	0.99	0.99	0.96	0.90	0.97	0.89	1.00
	*P*_*m*_(%)	99.2	107.5	105.6	106.3	109.2	109.7	101.8	97.4	75.5	110.9	78.1	100.0
	*α*	0.02	0.02	0.02	0.03	0.02	0.01	0.03	0.05	0.06	0.00	0.07	0.00
	Grade	1	2	2	2	2	2	1	2	4	3	4	1
350nm	*S*_*m*_	0.99	0.95	0.99	0.98	0.98	0.96	0.97	0.97	0.89	0.95	0.88	1.00
	*P*_*m*_(%)	99.3	109.2	108.8	107.5	112.0	105.7	104.2	94.6	93.5	114.5	97.0	100.0
	*α*	0.02	0.02	0.00	0.00	0.01	0.01	0.01	0.02	0.17	0.02	0.18	0.00
	Grade	1	2	2	2	3	2	1	2	4	3	4	1
Integrated	*S*_*m*_′	0.98	0.97	0.98	0.98	0.98	0.98	0.98	0.97	0.88	0.96	0.88	1.00
	*P*_*m*_′	98.8	106.8	108.5	107.7	112.0	106.4	104.3	95.0	92.0	114.6	96.0	100.0
	*α*′	0.01	0.04	0.02	0.02	0.02	0.02	0.02	0.03	0.17	0.06	0.17	0.00
	Grade	1	2	2	2	3	2	1	2	4	3	4	1

The integrated macro qualitative similarity $S_{m}^{'}$ was mainly evaluated for the similarity of the samples in chemical composition, and the integrated macro quantitative similarity $P_{m}^{'}$ and leveling coefficient *α*' were subsequently used to quantitatively gauge the similarity in the overall chemical contents with reference to the reference fingerprint. The quality grade (G) of each sample, as shown in Table 3, was assigned to each sample based on the $S_{m}^{'}$, $P_{m}^{'}$ and *α*' values according Table 3. The integrated qualitative similarity factors ($S_{m}^{'}$) of most of the samples are in the range of 0.94–0.98, except for S21 (0.88) and S23 (0.88). In comparison, there is a wider distribution of the integrated quantitative similarity factors $P_{m}^{'}$ (86.9–114.6%) with the lowest value for S9 (86.9%) and the highest for S22 (114.6%); however, all the samples have the $P_{m}^{'}$ values in the range 80%-120%, demonstrating that the overall chemicals contents are not significantly different among the 23 samples. In addition, the majority of the samples have the integrated *α*' values below 0.07, but S21 and S23 have significantly higher *α*' values (0.17). Both the integrated macro qualitative similarity factors ($S_{m}^{'}$) and leveling coefficient (*α*') values clearly point out that S21 and S23 are different from the rest samples both from the perspective of qualitative chemical composition and quantitative overall content.

The relationship between sample fingerprint fingerprints and quantitative content of the marker compounds was also investigated. Linear regression was performed using the macro quantitative similarity factors *P*_*m*_(%) calculated with 49 common peaks at 260 nm and the mean value of the content of the seven marker compounds (*P*_7*C*_%) for each sample (Table 2). A reasonable linear correlation was obtained between the quantitative similarity factors of the fingerprints and the actual contents of the seven marker compounds in the ISHI samples (*r* = 0.906). This relationship demonstrates that the selected marker compounds (UR, AD, CGA, CFA, CCA, LGR and LG) basically synchronously changed with the overall content of the ISHI preparation chmicals. Hence quantitative evaluation of the fingerprints by SQFM has the potential to replace the use of multiple marker compounds and provides a reliable and provides a feasible means to control the quality consistency of the ISHI preparations.

### SVM and PCA analysis

As the result presented in Table B in S3 File, the observed and predicted classification by SVM had a percent correct for 86.96%, indicating the efficient classification of SVM. The predicted probability by SVM (Table C in S3 File) shows that the 23 samples could be divided into two groups, namely, Cluster I with S1-S20 and S22 (P≥0.9), Cluster II with S21 and S23 (P<0.9).

**Fig 3:**
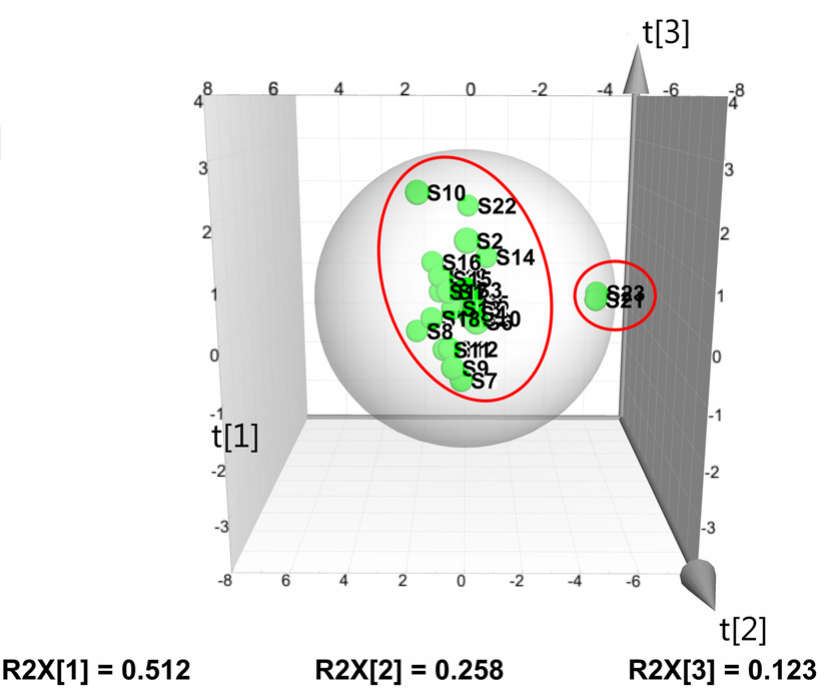
PCA score plot (given by SIMCA 13.0) of 23 batches of ISHI samples.

The PCA analysis was performed using a three-component model with a total variance of 89.3% explained (PC1 = 51.2%, PC2 = 25.8%, and PC3 = 12.3%). The PCA score plot in Fig 3 reveals that most samples fall into one cluster except S10, S21 and S23, but S21 and S23 are clearly in the same cluster. The PCA results are in a good agreement with the SVM analysis, showed a strong evidence that the quality of S21 and S23 may be different from the other samples. In fact the interesting *α*′ value of S10 was the highest among S10, S21 and S23 samples, which just better state why it is in the PCA score plot. When we look at all the evidences (SVM, PCA and SQFM analysis), we can come to the conclusion that S21 and S23 indeed are greatly different for *α*′ = 0.17.

### Antioxidant activity

#### Total antioxidant activity by off-line DPPH assay

Antioxidant activities have been demonstrated to be an effective *in vitro* measure to assess the biological activity of the ISHI preparations [19, 27]. The total antioxidant activities of the ISHI samples were assayed by the off-line DPPH method, where IC_50_ values were determined as shown in Table 2. The IC_50_ value represents the sample concentration required to scavenge 50% of DPPH radicals, and lower IC_50_ values indicate stronger antioxidant activities. A majority of the ISHI samples were found to possess acceptable antioxidant activities with an IC_50_ value less 5 mg/mL; however, S21 and S23 showed higher IC_50_ values (>5 mg/mL), indicating lower antioxidant activity.

#### Antioxidant activity prediction by PLS and OPLS models

To explore the relationship between the total antioxidant activity and the fingerprint constituents of the ISHI samples, both PLS and OPLS models were constructed using all the peak areas of 49 fingerprints at 260 nm and the antioxidant activity of the ISHI samples. It should be noted that the inverse of the IC_50_ values (1/ IC_50_) was selected as the *Y* variables to establish the models because lower IC_50_ values represent stronger antioxidant activity. After excluding the two outliers (S21 and S23) based on the *t*[1]-*t*[2] score plot, the remaining samples were divided randomly into a training set to establish the PLS (or OPLS) model and a test set to validate the model. The obtained calibration model of PLS and OPLS is expressed by Eq 11 and Eq 12, respectively. The linear regression models show that 24 peaks in the fingerprints had greater correlation with 1/IC_50_ in both PLS and OPLS model. Namely, 17 peaks, including peak 4, 5, 6, 7, 15, 16, 18, 20, 21, 24, 26, 33, 34, 36, 42, 45 and 47, were positively correlated; while 7 peaks, including peak 3, 12, 23, 25, 37, 41 and 43, were negatively correlated with 1/IC_50_.

$$Y_{PLS\left(1/IC_{50}\right)}=-94.6905-0.0670x_{3}+0.1004x_{4}+0.0797x_{5}+0.1252x_{6}+0.0773x_{7}-0.1304x_{12}+0.0918x_{15}+0.0691x_{16}+0.0590x_{18}+0.0738x_{20}+0.0605x_{21}-0.0724x_{23}+0.0821x_{24}-0.1650x_{25}+0.0926x_{26}+0.2214x_{33}+0.0700x_{34}+0.1760x_{36}-0.0889x_{37}-0.0960x_{41}+0.0494x_{42}-0.1238x_{43}+0.1250x_{45}+0.0800x_{47}$$ (11)

$$Y_{OPLS\left(1/IC_{50}\right)}=-97.9036-0.0783x_{3}+0.1106x_{4}+0.0841x_{5}+0.1228x_{6}+0.0823x_{7}-0.1216x_{12}+0.0909x_{15}+0.0706x_{16}+0.0488x_{18}+0.0866x_{20}+0.0656x_{21}-0.0636x_{23}+0.096x_{24}-0.1590x_{25}+0.0894x_{26}+0.2267x_{33}+0.0728x_{34}+0.1813x_{36}-0.0973x_{37}-0.0915x_{41}+0.0530x_{42}-0.1400x_{43}+0.1250x_{45}+0.0680x_{47}$$ (12)

**Fig 4:**
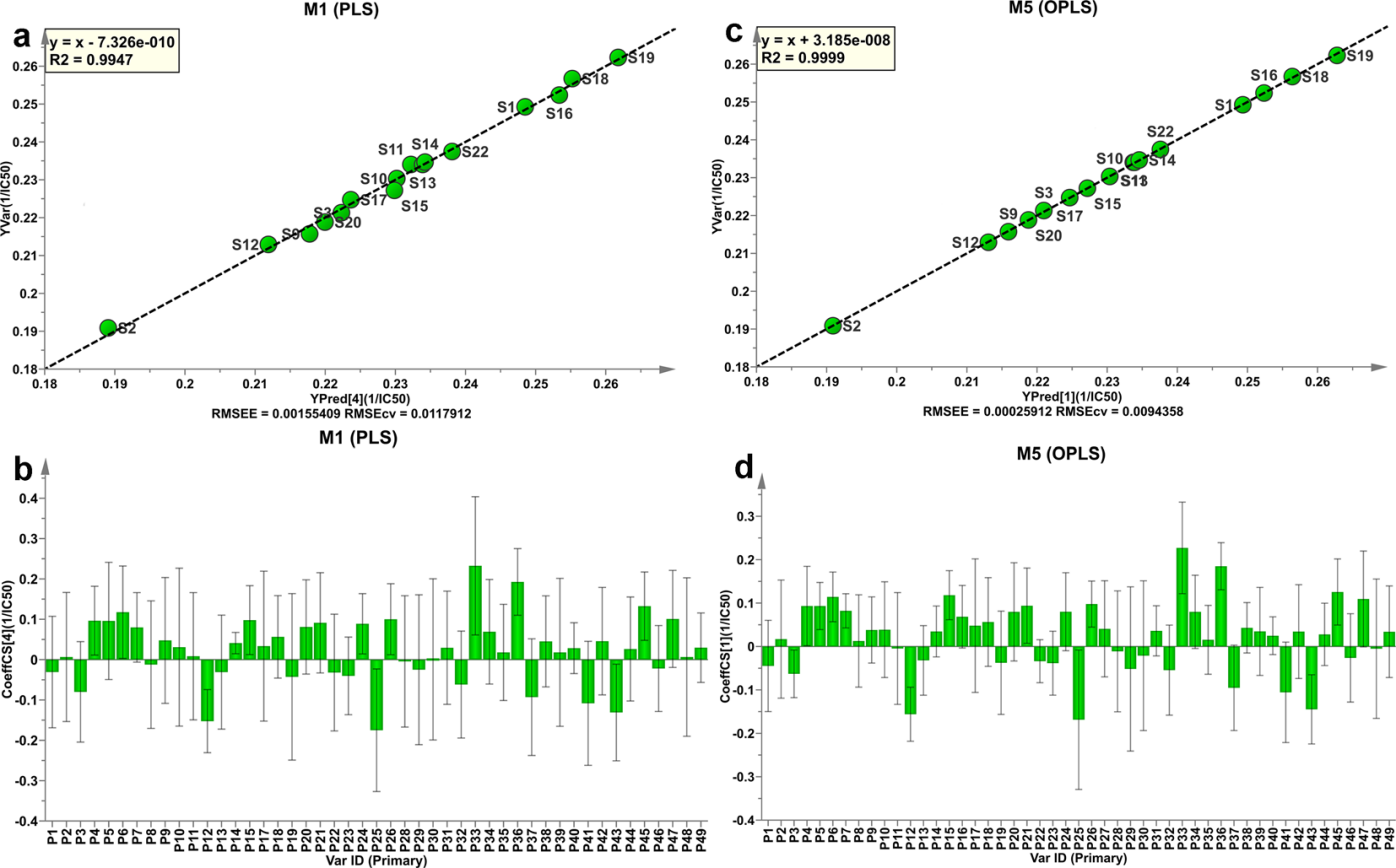
The *Y* observed versus *Y* predicted plots and coefficients plots for ISHIs: (a) *Y* observed versus *Y* predicted plot and (b) coefficients plot for PLS model. (c) *Y* observed versus *Y* predicted plot and (d) coefficients plot for OPLS model. The bars indicate 95% confidence intervals based on jack-knifing.

As shown in Fig 4A and 4C, the experimental 1/ IC_50_ values (*Y* Observed) are found to have a good correlation with the values predicted by the PLS or OPLS model with the explained variance (R^2^) of 99.47% and 99.99%, and the predictive ability (Q^2^) of 84.8% and 71.3% for the PLS and OPLS models, respectively. The root mean square error of estimation (RMSEE) and cross-validation procedure (RMSECV) are 0.0016 and 0.0118 for the PLS model, and 0.0003 and 0.0094 for the OPLS model, indicating that the OPLC model is a better fit than the PLS model.

The established PLS and OPLS models were validated using five samples that were not used for calibration. The desirable predictive power was demonstrated by the root mean square error of prediction (RMSEP) value of 0.0195 and 0.0178 for PLS and OPLS, respectively. The predicted and experimental 1/ IC_50_ values for both the PLS and OPLS models are presented in Table 4.

**Table 4:** Overview of the experimental and predicted values for total antioxidant activity of both the PLS and OPLS models.

PLS Model				OPLS Model				
Sample number	Predicted data	Experimental data	*RE*^c^ (%)	Sample number	Predicted data	Experimental data	*RE*^c^ (%)	Ratio^d^
S1^a^	0.248	0.249	-0.536	S1^a^	0.249	0.249	-0.019	28.4
S2^a^	0.189	0.191	-0.980	S2^a^	0.191	0.191	0.001	-935
S3^a^	0.222	0.221	0.359	S3^a^	0.221	0.221	-0.120	-3.0
S9^a^	0.218	0.216	1.065	S9^a^	0.216	0.216	0.141	7.5
S10^a^	0.230	0.230	0.052	S10^a^	0.230	0.230	0.052	1.0
S11^a^	0.232	0.234	-0.820	S11^a^	0.234	0.234	-0.096	8.6
S12^a^	0.212	0.213	-0.440	S12^a^	0.213	0.213	0.029	-15.3
S13^a^	0.234	0.234	0.008	S13^a^	0.234	0.234	-0.010	-0.8
S14^a^	0.234	0.235	-0.019	S14^a^	0.234	0.235	-0.019	1.0
S15^a^	0.230	0.227	1.208	S15^a^	0.227	0.227	-0.067	-17.9
S16^a^	0.253	0.252	0.278	S16^a^	0.252	0.252	-0.002	-140
S17^a^	0.224	0.225	-0.292	S17^a^	0.225	0.225	0.001	-218
S18^a^	0.255	0.257	-0.632	S18^a^	0.256	0.257	-0.099	6.4
S19^a^	0.262	0.262	-0.143	S19^a^	0.263	0.262	0.138	-1.0
S20^a^	0.220	0.219	0.572	S20^a^	0.219	0.219	0.043	13.2
S22^a^	0.234	0.237	-1.455	S22^a^	0.238	0.237	0.030	-48.6
S4^b^	0.199	0.202	-1.714	S4^b^	0.199	0.202	-1.714	1.0
S5^b^	0.219	0.244	-10.107	S5^b^	0.220	0.244	-9.764	1.0
S6^b^	0.223	0.243	-8.242	S6^b^	0.226	0.243	-7.194	1.1
S7^b^	0.238	0.227	4.659	S7^b^	0.239	0.227	5.133	0.9
S8^b^	0.189	0.217	-12.863	S8^b^	0.193	0.217	-10.878	1.2

^a^Used for the calibration model.

^b^Used for the prediction model.

^c^RE: relative error.

^d^The ratio of RE_PLS_/ RE_OPLS_.

#### Individual fingerprint components and antioxidant activity

The regression coefficients were also calculated for the scaled and centered *X*-variables (i.e., 49 common peaks) at 95% confidence interval to explore the relationship between individual fingerprint components and antioxidant activity. The regression coefficient plots in Fig 4B and 4D reveal that the majority of the fingerprint components (34 and 31 out of 49 peaks based on the PLS and OPLS models, respectively) appears to have a positive influence on the total antioxidant activity, and all the seven marker compounds have positive correlation coefficients in both the PLS and OPLS models. The regression coefficients also indicate that phenolic acids (CFA, CGA and CCA) have relatively higher antioxidant activity and flavonoid compounds (LGR and LG) lower antioxidant activity. Therefore, it is not surprising that S21 and S23 had weaker total antioxidant activities than the other samples (Table 2) because they contain less marker compounds (CFA, CGA, and CCA) with higher antioxidant activity, but more marker compounds (LGR and LG) with lower antioxidant activity.

#### Antioxidant activity by on-line DPPH assay

**Fig 5:**
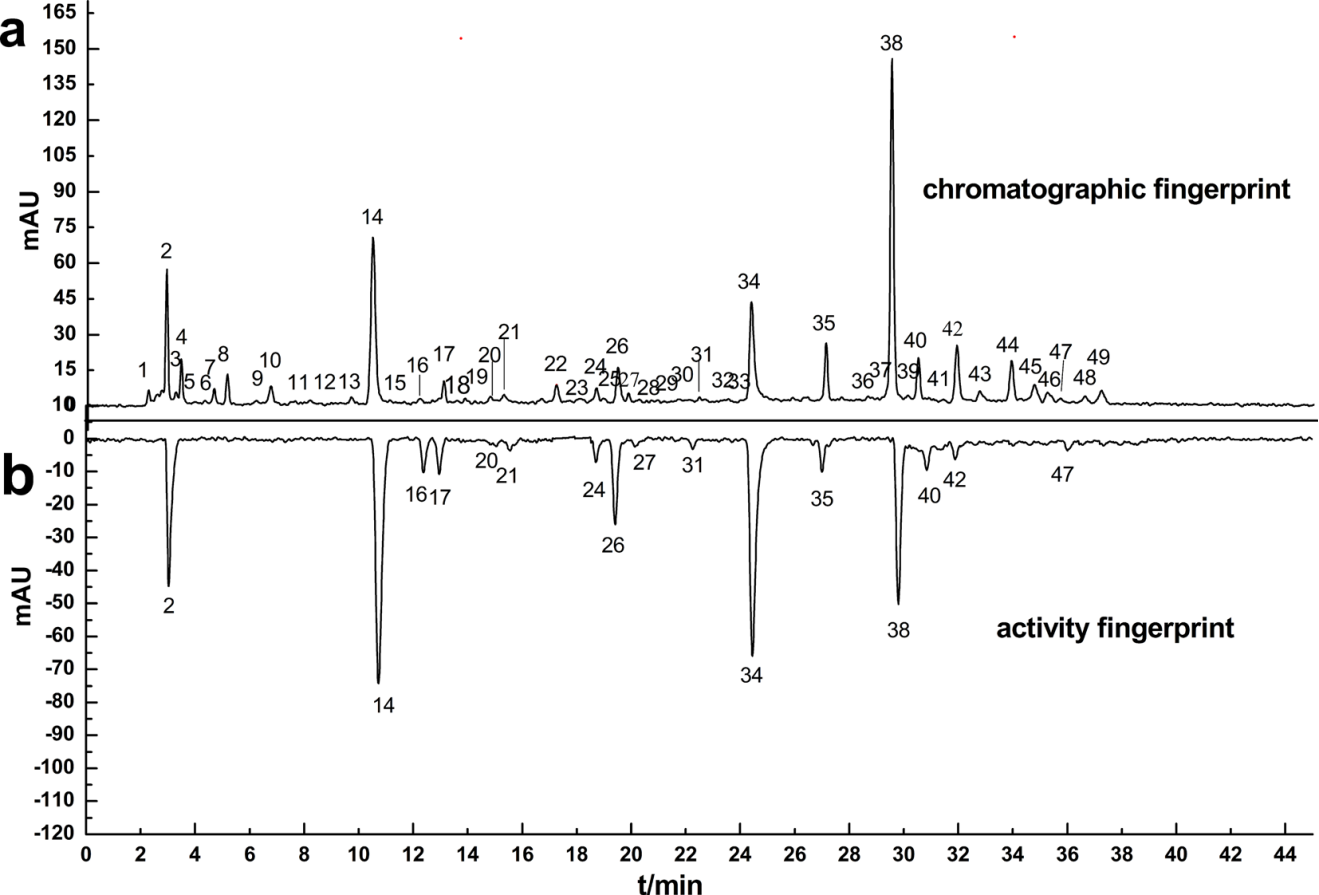
HPLC chromatograms of sample S5: detected at 260 nm (a) and 517nm (b, negative peaks indicating antioxidant activity). The identified peaks include: 4-UR, 10-AD, 24-CGA, 26-CFA, 34-CCA, 38-LGR and 40-LG.

The antioxidant activities of the ISHI samples were also determined by the on-line HPLC-DAD-DPPH method. Fig 5A and 5B display the chromatographic fingerprint of Sample 5 (S5) with 49 common peaks detected at 260 nm and antioxidant activity fingerprint at 517 nm, respectively. The negative peaks in the activity fingerprint indicate that these components have free radical scavenging activity. Among the seven marker compounds identified in the chromatographic fingerprint, the antioxidant activity was clearly observed for five marker compounds (CGA-24, CFA-26, CCA-34, LGR-38 and LG-40 in Fig 5B), which was consistent with their positive correlation to the antioxidant activity based on the PLS and OPLS models (Fig 4B and 4D). In comparison, the antioxidant activity was not directly observed for UR-4 in the activity fingerprint possibly due to the chromatographic resolution of the activity peaks (peak 2 in Fig 5B) and/or the sensitivity of the online DPPH method. Uridine elutes closely to an unknown compound (peak 2 in the chromatographic fingerprint). It is possible that the UR peak in the activity fingerprint was not resolved from the activity peak 2. In addition, UR is the least abundant marker compound; therefore, the UR activity peak may not be detected due to the sensitivity of the online DPPH method. The antioxidant activity was not detected for AD as shown in Fig 5B most likely due to its very low (if any) antioxidant activity. The online DPPH assay data suggests that the antioxidant activity of the ISHI samples might be attributed to the presence of phenolic acid [36] and flavonoid components, but not to nucleoside components. In addition to the marker compounds, other unknown components in the ISHI sample also showed significant antioxidant activities, for example, peak 2, 14, 16 and 17 (Fig 5B). In contrast, other unknown components (peak 8 and 44) detected in the chromatographic fingerprint did not show any antioxidant activity in the activity fingerprint in consistence with their negative regression coefficients calculated from the PLS model (Fig 4B). The other 22 samples showed similar antioxidant activity fingerprints (not shown). The online antioxidant activity assay has a clear advantage over the offline assay method in that the individual contribution to the total antioxidant activity by each chemical component can be determined, and the unknown compounds with significant antioxidant activity can be identified for further structural elucidation.

## Conclusions

A multi-prong approach including chemometric methods (SVM and PCA), quantitative fingerprint evaluation, and antioxidant activity assay was employed to evaluate the quality consistency of 23 batches of the ISHI injectable preparations. Clustering based on SVM and PCA is able to identify two samples (S21 and S23) that are not similar to the other samples. Simultaneous analysis of the seven known marker compounds provides the quantitative information which helps to explain the difference observed in the SVM and PCA patterns. S21 and S23 had much lower content in one marker compound (AD), but higher content in two other marker compounds (LGR and LG) than the other samples. The characteristic fingerprints generated at multiple wavelengths further disclose the qualitative and quantitative difference in the chemical composition of these samples when evaluated with SQFM. In addition to the known marker compounds, other unknown components of the ISHI samples were evaluated for similarity based on their fingerprints. Again S21 and S23 are shown to be different from the other samples by both the qualitative similarity factor ($S_{m}^{'}$) and quantitative similarity factor (particularly *α*') in consistence with the chemometric and quantitative marker compound analysis. Moreover the total antioxidant activities of the ISHI samples were determined by the offline DPPH assay and a correlation was also established between the antioxidant activity and the total amount of the marker compounds based on the PLS and OPLS models. The online DPPH assay further elucidates individual contribution of the chemical components to the total antioxidant activity, and provides a solid explanation why S21 and S23 had lower antioxidant activity. Therefore, this multi-prong approach provides a holistic approach to evaluate the quality consistency of the complex multi-component TCM and their preparations.

Although the chemometric methods (SVM and PCA) are able to identify different samples based on clustering and patterns, it is very difficult to apply these methods to the quality control of the TCM and herbal preparations in a manufacturing environment since clustering or pattern recognition requires the comparison of a large number of samples. Multiple marker compounds could be used, in theory, for quality control purpose; however, the biological or pharmacological effects of the marker compounds must be known. And it is also difficult to perform quantitation of multiple marker compounds in a fast-paced QC laboratory. In comparison, the SQFM has significant advantages for the quality control purpose. First, the qualitative similarity factor *S*_*m*_ can reveal the difference in chemical composition of the samples, similar to the SVM and PCA methods. Second, the quantitative measures (i.e., the quantitatively similarity factor *P*_*m*_) are also be to evaluate the difference in the overall content of the fingerprints. Finally the leveling coefficient *α* is a sensitive parameter to subdivide the category of samples. Once the standard prescription are set (i.e. the reference fingerprint is determined before one of sample determined), the two similarity factors (*S*_*m*_, *P*_*m*_) and one leveling coefficient (*α*) can be effectively briefly used for the product release of a single lot for all TCM or herbal medicine, which cannot be done using the chemometric methods that need so many samples.

## Supporting Information

S1 File The values of mobile phase (MP: MP1~MP4) conditions and gradient elution programs (GEP: GEP1~GEP3). **MP1**: aqueous solution containing 1.0% (v/v) glacial acetic acid (A) and acetonitrile containing 1.0% (v/v) glacial acetic acid (B); **MP2**: aqueous solution containing 10 mM sodium dihydrogen phosphate (A) and acetonitrile containing 1.0% (v/v) glacial acetic acid (B); **MP3**: aqueous solution containing 5 mM citric acid and 10 mM sodium dihydrogen phosphate (A) and acetonitrile containing 1.0% (v/v) glacial acetic acid (B); **MP4**: aqueous solution containing 6 mM citric acid and 10 mM sodium dihydrogen phosphate (A) and acetonitrile containing 1.0% (v/v) glacial acetic acid (B); **GEP1**: 0–5% B at 0-10min, 5–12% B at 10–25 min, 12–17% B at 25–40 min, 17–25% B at 40–60 min; **GEP2**: 0–2% B at 0–5 min, 2–5% B at 5–10 min, 5–9% B at 10–16 min, 9–15% B at 16–25 min, 15–18% B at 25–40 min, 18–25% B at 40–60 min; **GEP3**: 0–3% B at 0–5 min, 3–7% B at 5–10 min, 7–10% B at 10–16 min, 10–17% B at 16–25 min, 17–19% B at 25–30 min, 19–20% B at 30–40 min, 20–25% B at 40–60 min.
(DOC)

S2 File The list of the main abbreviations: **ISHI**, Ixeris sonchifolia (Bge.) Hance Injectable;**SQFM**, systematic quantitative fingerprint method;**SVM**, Support vector machine;**PCA**, principal component analysis;**DPPH**, 2,2-diphenyl-1-picryldrazyl;**PLS**, partial least squares;**OPLS**, orthogonal projection to latent structures;**TCM**, Traditional Chinese Medicine;**UR**, Uridine;**AD**, Adenosine;**CGA**, Chlorogenic acid;**CFA**, Caffeic acid;**CCA**, Chicoric acid;**LGR**, Luteolin-7-β-D-glucuronide;**LG**, Luteolin-7-glucoside;**MP**, mobile phase;**GEP**, gradient elution program.
(DOC)

S3 File **Table A in S3 File.** The quality grades classified by SQFM. **Table B in S3 File.** Comparing the observed and predicted classification by SVM. **Table C in S3 File.** Overview of the classification results of SVM.
(DOCX)
