# Supplementary material for: Holistic Evaluation of Quality Consistency of Ixeris sonchifolia (Bunge) Hance Injectables by Quantitative Fingerprinting in Combination with Antioxidant Activity and Chemometric Methods
Source: PLoS One. 2016 Feb 12;11(2):e0148878. doi: 10.1371/journal.pone.0148878 (PMC4752467; doi:10.1371/journal.pone.0148878)
Supplement: S1 File — MP1: aqueous solution containing 1.0% (v/v) glacial acetic acid (A) and acetonitrile containing 1.0% (v/v) glacial acetic acid (B); MP2: aqueous solution containing 10 mM sodium dihydrogen phosphate (A) and acetonitrile containing 1.0% (v/v) glacial acetic acid (B); MP3: aqueous solution containing 5 mM citric acid and 10 mM sodium dihydrogen phosphate (A) and acetonitrile containing 1.0% (v/v) glacial acetic acid (B); MP4: aqueous solution containing 6 mM citric acid and 10 mM sodium dihydrogen phosphate (A) and acetonitrile containing 1.0% (v/v) glacial acetic acid (B); GEP1: 0–5% B at 0-10min, 5–12% B at 10–25 min, 12–17% B at 25–40 min, 17–25% B at 40–60 min; GEP2: 0–2% B at 0–5 min, 2–5% B at 5–10 min, 5–9% B at 10–16 min, 9–15% B at 16–25 min, 15–18% B at 25–40 min, 18–25% B at 40–60 min; GEP3: 0–3% B at 0–5 min, 3–7% B at 5–10 min, 7–10% B at 10–16 min, 10–17% B at 16–25 min, 17–19% B at 25–30 min, 19–20% B at 30–40 min, 20–25% B at 40–60 min. (DOC) [file pone.0148878.s001.doc]

**Supporting information 1. The values of mobile phase (MP: MP1～MP4) conditions and gradient elution programs (GEP: GEP1～GEP3).** **MP1**: aqueous solution containing 1.0% (v/v) glacial acetic acid (A) and acetonitrile containing 1.0% (v/v) glacial acetic acid (B); **MP2**: aqueous solution containing 10 mM sodium dihydrogen phosphate (A) and acetonitrile containing 1.0% (v/v) glacial acetic acid (B); **MP3**: aqueous solution containing 5 mM citric acid and 10 mM sodium dihydrogen phosphate (A) and acetonitrile containing 1.0% (v/v) glacial acetic acid (B); **MP4**: aqueous solution containing 6 mM citric acid and 10 mM sodium dihydrogen phosphate (A) and acetonitrile containing 1.0% (v/v) glacial acetic acid (B); **GEP1**: 0-5% B at 0-10min, 5-12% B at 10-25 min, 12-17% B at 25-40 min, 17-25% B at 40-60 min; **GEP2**: 0-2% B at 0-5 min, 2-5% B at 5-10 min, 5-9% B at 10-16 min, 9-15% B at 16-25 min, 15-18% B at 25-40 min, 18-25% B at 40-60 min; **GEP3**: 0-3% B at 0-5 min, 3-7% B at 5-10 min, 7-10% B at 10-16 min, 10-17% B at 16-25 min, 17-19% B at 25-30 min, 19-20% B at 30-40 min, 20-25% B at 40-60 min.
