# Supplementary material for: Holistic Evaluation of Quality Consistency of Ixeris sonchifolia (Bunge) Hance Injectables by Quantitative Fingerprinting in Combination with Antioxidant Activity and Chemometric Methods
Source: PLoS One. 2016 Feb 12;11(2):e0148878. doi: 10.1371/journal.pone.0148878 (PMC4752467; doi:10.1371/journal.pone.0148878)
Supplement: S2 File — ISHI, Ixeris sonchifolia (Bge.) Hance Injectable;SQFM, systematic quantitative fingerprint method;SVM, Support vector machine;PCA, principal component analysis;DPPH, 2,2-diphenyl-1-picryldrazyl;PLS, partial least squares;OPLS, orthogonal projection to latent structures;TCM, Traditional Chinese Medicine;UR, Uridine;AD, Adenosine;CGA, Chlorogenic acid;CFA, Caffeic acid;CCA, Chicoric acid;LGR, Luteolin-7-β-D-glucuronide;LG, Luteolin-7-glucoside;MP, mobile phase;GEP, gradient elution program. (DOC) [file pone.0148878.s002.doc]

**Supporting information 2. The list of the main abbreviations:**

**ISHI**, *Ixeris sonchifolia* (Bge.) Hance Injection;

**SQFM**, systematic quantitative fingerprint method;

**HCA**, hierarchical cluster analysis;

**PCA**, principal component analysis;

**DPPH**, 2,2-diphenyl-1-picryldrazyl;

**PLS**, partial least squares;

**OPLS**, orthogonal projection to latent structures;

**TCM**, Traditional Chinese medicine;

**UR**, Uridine;

**AD**, Adenosine;

**CGA**, Chlorogenic acid;

**CFA**, Caffeic acid;

**CCA**, Chicoric acid;

**LGR**, Luteolin-7-β-D-glucuronide;

**LG**, Luteolin-7-glucoside;

**MP**, mobile phase ;

**GEP**, gradient elution program.
