# Supplementary material for: Holistic Evaluation of Quality Consistency of Ixeris sonchifolia (Bunge) Hance Injectables by Quantitative Fingerprinting in Combination with Antioxidant Activity and Chemometric Methods
Source: PLoS One. 2016 Feb 12;11(2):e0148878. doi: 10.1371/journal.pone.0148878 (PMC4752467; doi:10.1371/journal.pone.0148878)
Supplement: S3 File — Table A in S3 File. The quality grades classified by SQFM. Table B in S3 File. Comparing the observed and predicted classification by SVM. Table C in S3 File. Overview of the classification results of SVM. (DOCX) [file pone.0148878.s003.docx]

**Table S1.** The quality grades classified by SQFM.

| Grade | 1 | 2 | 3 | 4 | 5 | 6 | 7 | 8 |
| --- | --- | --- | --- | --- | --- | --- | --- | --- |
| *Sm≥* | 0.95 | 0.90 | 0.85 | 0.80 | 0.70 | 0.60 | 0.50 | ＜0.50 |
| *Pm*(%)∈ | 95～105 | 90～110 | 80～120 | 75～125 | 70～130 | 60～140 | 50～150 | 0～∞ |
| *α*≤ | 0.05 | 0.10 | 0.15 | 0.20 | 0.30 | 0.40 | 0.50 | ＞0.50 |
| Quality | Best | Better | Good | Fine | Moderate | Common | Defective | Inferior |

**Table S2.** Comparing the observed and predicted classification by SVM.

| Observed | Predicted | | |
| --- | --- | --- | --- |
|  | -1 | 1 | Percent Correct |
| -1 | 18 | 0 | 100% |
| 1 | 3 | 2 | 60% |
| Overall Percentage | 91.3% | 8.7% | 86.96% |

**Table S3.** Overview of the classification results of SVM.

| Sample | Quality Grade^a^ | Observed Class^b^ | Predicted Class^c^ | Predicted Probability |
| --- | --- | --- | --- | --- |
| S1 | 2 | -1 | -1 | 0.9654 |
| S2 | 2 | -1 | -1 | 0.9516 |
| S3 | 2 | -1 | -1 | 0.9442 |
| S4 | 1 | -1 | -1 | 0.9387 |
| S5 | 1 | -1 | -1 | 0.9351 |
| S6 | 2 | -1 | -1 | 0.9302 |
| S7 | 1 | -1 | -1 | 0.9474 |
| S8 | 2 | -1 | -1 | 0.9782 |
| S9 | 3 | 1 | -1 | 0.9562 |
| S10 | 2 | -1 | -1 | 0.9769 |
| S11 | 1 | -1 | -1 | 0.9646 |
| S12 | 2 | -1 | -1 | 0.9609 |
| S13 | 1 | -1 | -1 | 0.9608 |
| S14 | 2 | -1 | -1 | 0.9302 |
| S15 | 2 | -1 | -1 | 0.9645 |
| S16 | 2 | -1 | -1 | 0.9713 |
| S17 | 3 | 1 | -1 | 0.9633 |
| S18 | 2 | -1 | -1 | 0.9722 |
| S19 | 1 | -1 | -1 | 0.9664 |
| S20 | 2 | -1 | -1 | 0.9350 |
| S21 | 4 | 1 | 1 | 0.7365 |
| S22 | 3 | 1 | -1 | 0.9467 |
| S23 | 4 | 1 | 1 | 0.7365 |

^a^ The integrated grade from Table 3.

^b^ If grade≤2, make y=-1; if grade＞2, make y=1.

^c^ Predicted class by SVM.
